# Diet, Microbiota and Brain Health: Unraveling the Network Intersecting Metabolism and Neurodegeneration

**DOI:** 10.3390/ijms21207471

**Published:** 2020-10-10

**Authors:** Francesco Gentile, Pietro Emiliano Doneddu, Nilo Riva, Eduardo Nobile-Orazio, Angelo Quattrini

**Affiliations:** 1 Experimental Neuropathology Unit, Institute of Experimental Neurology, Division of Neuroscience, San Raffaele Scientific Institute, 20132 Milan, Italy; francesco.gentile@humanitas.it (F.G.); riva.nilo@hsr.it (N.R.); 2 Neuromuscular and Neuroimmunology Service, Humanitas Clinical and Research Institute IRCCS, 20089 Milan, Italy; pietro.doneddu@humanitas.it (P.E.D.); eduardo.nobile_orazio@humanitas.it (E.N.-O.); 3 Department of Neurology, San Raffaele Scientific Institute, 20132 Milan, Italy; 4 Department of Medical Biotechnology and Translational Medicine, University of Milan, 20122 Milan, Italy

**Keywords:** dysbiosis, diet, metabolism, Alzheimer’s disease, Parkinson’s disease, amyotrophic lateral sclerosis

## Abstract

Increasing evidence gives support for the idea that extra-neuronal factors may affect brain physiology and its predisposition to neurodegenerative diseases. Epidemiological and experimental studies show that nutrition and metabolic disorders such as obesity and type 2 diabetes increase the risk of Alzheimer’s and Parkinson’s diseases after midlife, while the relationship with amyotrophic lateral sclerosis is uncertain, but suggests a protective effect of features of metabolic syndrome. The microbiota has recently emerged as a novel factor engaging strong interactions with neurons and glia, deeply affecting their function and behavior in these diseases. In particular, recent evidence suggested that gut microbes are involved in the seeding of prion-like proteins and their spreading to the central nervous system. Here, we present a comprehensive review of the impact of metabolism, diet and microbiota in neurodegeneration, by affecting simultaneously several aspects of health regarding energy metabolism, immune system and neuronal function. Advancing technologies may allow researchers in the future to improve investigations in these fields, allowing the buildup of population-based preventive interventions and development of targeted therapeutics to halt progressive neurologic disability.

## 1. Introduction

Neurodegenerative disorders are characterized by age-related dysfunction in several cognitive and motor domains, loss of self-sufficiency and death. In most cases, patients present sporadically, even though a family history may be present in about 5–20% of the diseased population, such as in Alzheimer’s disease (AD), Parkinson’s disease (PD) and amyotrophic lateral sclerosis (ALS). The role of genetics has been increasingly recognized in recent years, showing that mutations and/or rare variants in specific genes contribute to the development of disease susceptibility [1].

Environmental factors also participate to the pathogenesis of neurodegenerative disorders, influencing disease risk and course in different manners [2]. Smoking is an important risk factor for vascular dementia and AD, while it appears protective against PD. Occupational exposure to contaminants such as pesticides or heavy metals has also been associated to increased risk of neurodegenerative disorders [3,4,5].

Nutrition is an important contributor of long-life exposure to environmental factors and the modern diets consumed in Western countries, enriched in fat and sugars, are largely responsible for the high prevalence of metabolic disorders, such as obesity, type 2 diabetes (T2D) and metabolic dysfunction-associated fatty liver disease (MAFLD). Dietary patterns and specific nutrients have been associated with neurodegenerative diseases in humans, as shown in large prospective population studies and experimental models. The microbial community dwelling in the gut, also known as the gut microbiota (GM), exerts numerous functions for gut homeostasis and host health [6], such as metabolism of undigested nutrients, supply of beneficial microbial metabolites, defense against enteric pathogens and maturation of the immune system. More than 1000 species inhabit the gut, mainly classified in five phyla: *Bacteroidetes* and *Firmicutes* dominate about 90% of the microbial community, whereas *Actinobacteria*, *Proteobacteria* and *Verrucomicrobia* are relatively minor constituents. Nutrition and microbiota are closely related to each other, as dietary habits affect colonization, maturation and changes to the microbiome throughout life [7,8]. Recent evidence showed that the GM participates in brain physiology and disruption in its composition, leading to dysbiosis, may contribute to neurodegeneration. Diverse signaling pathways are elicited by harmful nutrients and microbes, such as energy metabolism, oxidative stress, mitochondrial function and neuroinflammation. Furthermore, they may affect cellular function through epigenetic mechanisms, such as DNA methylation, histone modifications and non-coding RNA expression, stably influencing the gene expression profile of cells for long periods. Such events may show some degree of reversibility, although permanent changes may occur in critical life periods, such as during childhood or mid-adulthood, affecting the risk of age-related human disorders. For instance, the diet consumed during young age may predict the lifetime risk of diabetes, cardiovascular disease and overall mortality [9], which could be spread to the offspring, potentially revealing a trans-generational heritability of dietary effects [10]. 

Here, we aim to review the mechanisms linking metabolism, diet and microbiota to brain health. Both direct and indirect effects on neuronal signaling and survival will be discussed, unraveling the bidirectional communication between the gut and the brain through the neuroendocrine axis, the immune system and systemic circulation of nutrients and metabolites. Then, we will analyze the role of these three factors in the development of neurodegenerative diseases, focusing on AD, PD and ALS to highlight fields of translational research and applications to clinical practice.

## 2. Research Method and Data Collection

This research carried out a systematic search on PubMed and Google Scholar databases updated until September 6th, 2020. The search keywords were: “Alzheimer”, “Parkinson”, “Amyotrophic Lateral Sclerosis”, “Neurodegeneration”, “Brain disorders”, “Brain health” in combination with “Diet”, “Nutrients”, “Nutrigenomics”, “Nutrigenetics”, “Metabolism”, “Obesity”, “Diabetes, “NAFLD”, “Cholesterol”, “Lipids”, “PUFAs”, “Insulin”, “Microbiota”, “Gut-brain axis”, and “Probiotics”. 

F.G. conducted the primary research and screened titles and abstracts of search outputs. Only articles published in were included. In the end, 387 peer-reviewed research articles based on experimental-based and clinical data (mainly prospective studies) were collected, including 11 systematic reviews and 13 meta-analysis. Among them, 96 research papers were reviewed from 6 major management science publishers, namely *Nature, Cell, Neurology, Annals of Neurology, JAMA Neurology and Acta Neuropathologica.* Critical aspects and controversial results were spotlighted and critically discussed in an attempt to provide inspiration for future research directions.

## 3. Nutrients, Microbiota and Brain Health

The central nervous system (CNS) is a highly energy demanding organ, as it uses about 20% of the total oxygen and glucose consumed by the body, despite representing only 2% of the total body mass. Neurons heavily rely on glucose as the main energy substrate, but in stressful conditions, other resources, such as ketone bodies and lactate, provided by glial cells, may be used. Fatty acids (FA) are poorly used by the CNS as a fuel due to a low expression of the β-oxidation enzyme machinery, an evolutionarily acquired feature necessary to limit excessive oxygen consumption and consequent reactive oxygen species generation in mitochondria generally associated with FA catabolism [11]. Furthermore, the CNS has a limited ability to build internal energy stores, as only astrocytes have been shown to synthesize glycogen in small amounts [12]. Cholesterol is essential for brain function. It is involved in cell maintenance, neuronal transmission, and synaptic formation. Its metabolism in the CNS relies on local de novo synthesis and catabolism, as the blood–brain barrier (BBB) blocks the passage of diet-derived cholesterol into the CNS [13]. 

Thus, to maintain a constant delivery of energy substrates for neuronal activity, the CNS engages in intensive crosstalk with organs involved in metabolism, such as the gut, adipose tissue and liver, regulating several functions such as food behavior, hormonal status and commence of adaptive responses to dietary changes [14]. Due to its metabolic setting, the maintenance of glucose homeostasis is essential for proper neuronal functioning. Receptors for insulin and insulin-like growth factor-1 (IGF-1) are present throughout the CNS, mostly concentrated on the hypothalamus and hippocampus, where local production of these hormones has also been demonstrated, especially during growth. Insulin and IGF-1 exert an important role in neuronal development and survival by stimulating synaptic plasticity and long-term potentiation, which aid in learning and memory. Interestingly, insulin modulates phosphorylation of tau protein, supporting a potential involvement of insulin metabolism in AD [15]. Furthermore, fibroblast growth factor 21 (FGF21), a hepatocyte-derived hormone, signals protein and glucose status to the brain, allowing the refinement of food choice and metabolism according to dietary changes [16,17]. On the other hand, CNS insulin sensitivity modulates adiposity and body fat accumulation [18]. Along the brain-periphery signaling network, diet and microbiota deeply influence these communication pathways through several mechanisms. 

### 3.1. Diet-CNS Interactions

Cognition and behavior are tightly connected to the nutritional state of the organism. Regulation in many neural- and nutrition-related genes underwent strong positive selection during human evolution, representing an important step forward in the separation from primates along the evolutionary timeline and contributing to the high complexity of human communication and habits [19]. 

Besides its basic function as an energy substrate, the type and composition of diet during early life has important long-lasting effects on brain function. Breastfeeding is associated with better neural development in childhood [20] and its effects persist during adulthood, as the better cognitive performance of breastfed progeny associates with higher educational status and income [21]. Nonetheless, these effects may be viable only within precise time windows during growth. Indeed, consumption of 2′-fucosyllactose, a component of human milk, from 1 month after birth predicted better cognition at 24 months, but this effect was lost if supplied from 6 months of age [22]. 

Long-term effects may be explained by the ability of nutrients to exert stable epigenetic changes on neurons, critical for proper development of the nervous system. Omega-3 polyunsaturated FAs (ω-3 PUFA), including docosahexaenoic acid (DHA) and eicosapentaenoic acid (EPA), are essential components of neuronal cells’ membrane, affecting its composition and fluidity. Examples of food enriched in ω-3 PUFAs are fish, evo oil, walnuts and soybeans. High concentrations of DHA and EPA are present in the nervous tissue, representing 40% of the lipid content in neuronal membranes, and their accumulation in the brain during gestational and perinatal age is important for vision, memory, and behavior. Besides their structural role, perinatal ω-3 PUFA-enriched diet induces wide ranging changes in the brains of rats at the transcriptional level, affecting the expression of genes such as transthyretin, α-synuclein, and calmodulins, important for synaptic plasticity, hippocampal neurogenesis and learning [23]. Furthermore, ω-3 PUFAs exhibit anti-inflammatory effects. For these reasons, ω-3 PUFA supplementation, mainly through breastfeeding, is recommended in pregnant women and newborns [24]. 

Other micronutrients contribute to brain development and function. Flavonoids are plant-extracted polyphenols, which sustain spatial working memory by increasing hippocampal levels of brain-derived neurotrophic factor (BDNF), important for hippocampal neurogenesis [25,26]. Dietary choline supports fetal development during pregnancy by increasing neuronal proliferation and brain angiogenesis [27]. It also serves as a substrate for the production of the neurotransmitter acetylcholine and as a methyl-group donor, contributing to epigenetic modifications such as DNA and histone methylation. The 1958G>A polymorphism in the methylenetetrahydrofolate dehydrogenase gene (*MTHFD1*), which encodes for an enzyme involved in folate metabolism, decreases the availability of methyltetrahydrofolate, creating an extra demand of alternative methyl-group donors, such as choline, for the formation of methionine [28]. In a scenario of low choline diet, individuals carrying this SNP show a higher chance of developing fatty liver and muscle injury [28]. Finally, minerals and vitamins also exert numerous effects on neuronal signaling and communication. B vitamins are important for fiber myelination and neuronal survival, while vitamin E is a powerful antioxidant, supporting mitochondrial function in cells [29].

Despite several studies that analyzed isolated nutrients, investigating the effects of dietary patterns yields more reliable results as it closely reproduces natural human practices. The Mediterranean Diet (MeD) roots from southern Europe eating habits and is based on high consumption of vegetables, fruits, nuts and whole grains, moderate consumption of dairy products and limited amounts of meat and saturated fat. Besides its known effects in cardiovascular and cancer prevention, adherence to MeD demonstrated protection against a wide range of CNS diseases, including stroke, mild cognitive impairment and AD [30]. The results of functional tests are coherent with longitudinal neuroimaging studies, which showed that a healthy diet is associated with larger hippocampal volumes compared to individuals consuming a Western diet [31,32]. Caloric restriction has been also demonstrated to prolong lifespan and protects against neurodegeneration [33]. Reduction in calories stimulates a mild chronic stress response in neurons, which favors an increased production of neurotrophic factors, such as BDNF, and chaperones, protective against neuronal death and protein aggregation [33]. 

### 3.2. Gut Microbiota-CNS Interactions

**Figure 1:**
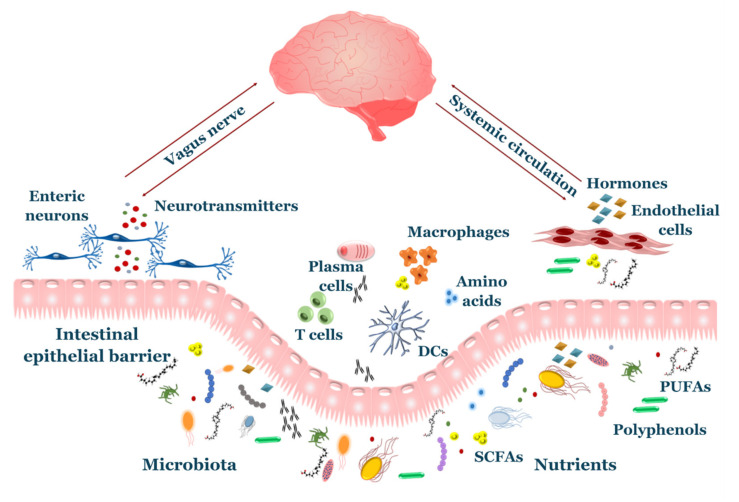
Overview of gut–brain axis. The gut and brain communicate through an intensive crosstalk involving neuroendocrine axis, circulating metabolites and immune system. Nutrients and microbial products pass through the intestinal epithelial barrier where they participate on enterocytes’ physiology and drive behavior of immune cells. The enteric nervous system uses numerous signals to sense the gut environment, including neurotransmitters produced by microbiota. Afferent fibers (vagus nerve/sympathetic nerves) transmit these signals to the central nervous system (CNS). Gut-derived hormones and metabolites are released to the systemic circulation and reach the brain.

The symbiotic relationship between gut microbiota and the brain, also known as the “microbiota-gut-brain axis”, exerts an important role in several aspects of brain health, such as development, function, and senescence. It is based on a continuous bi-directional flux of molecular signals using two main different routes: neural pathways and the systemic circulation (Figure 1). Viscero-fugal fibers make up 80% of the total vagus nerve, allowing the brain to constantly sense the gut environment. Indeed, neurotransmitters such as serotonin, dopamine and γ-aminobutyric acid (GABA) are produced by microbiota and bind the sensory neurons of the enteric nervous system (ENS), sending afferent impulses through the vagus nerve and sympathetic/parasympathetic pathways [34]. Gut inflammation induced by bacterial pathogens elicits activation of the vagal sensory ganglia and nucleus tractus solitarius in the brainstem, providing a method of early warning to the brain during infections [35]. Interestingly, some studies highlighted the existence of lateralized functions of the vagal pathway, such that the right vagus nerve is involved in stimulation of neural circuits responsible for diet-induced reward behavior [36], while the left vagus nerve takes part to a liver–brain–gut neural arc responsible for proper differentiation and maintenance of T regulatory (T_reg_) cells in the gut [37]. 

Neurotransmitters produced by GM also exert other functions outside of the nervous system with indirect effects of neuronal function. For instance, serotonin and dopamine affect immune system response [38,39], while GABA plays a role in the local defense against bacterial translocation [40]. The gut releases significant amounts of hormones, peptides and microbial metabolites, such as short chain fatty acids (SCFAs), secondary bile acids, tryptophan- and polyphenol-derived products, which exerts important effects on neuronal function and survival. Most of these compounds cross the BBB, including SCFAs, which exploit active membrane transporters on the endothelium to reach the CNS [41]. Conversely, the CNS also sends efferent responses to the gut, regulating motility, mucus secretion, barrier integrity and visceral sensitivity [42].

The GM exerts a broad role in the regulation of behavior and cognition. Germ-free (GF) animals show reduced anxiety [43,44], but impaired social development, providing links with neurodevelopmental and neuropsychiatric illnesses, such as autism spectrum disorders and schizophrenia [45]. Furthermore, gut bacteria participate in the proper development of non-spatial memory and fear extinction learning in mice, revealing targeted actions also towards the hippocampus [46] and prefrontal cortex [47], respectively. Food choice is also affected by GM, as O’Donnell et al. showed the ability of microbes to shape host’s dietary preferences towards food enriched in those specific bacteria, thus ensuring their survival and maintenance in the gut [48]. Microbial-derived metabolites stimulate the secretion of anorexigenic and incretin hormones, thus affecting appetite sensation and glucose metabolism after a meal [49]. Recent evidence revealed that GM might influence behavior in humans as well. Indeed, GM composition is associated with emotionality and fear reactivity in infants [50], important traits that have been shown to predict the risk of anxiety and depression. Furthermore, gut bacteria may model human personality traits, with sociability being associated with higher GM diversity, while anxiety and stress is related to reduced diversity [51]. 

GF mice develop gross and ultrastructural alterations in the amygdala and hippocampus, with the latter displaying shorter and less branched dendritic trees [52]. Microbiota also affects neuronal proliferation, as post-natal administration of antibiotics reduces hippocampal neurogenesis [53]. Among candidate mediators for this effect, sodium butyrate, a SCFA, restores antibiotic-induced impairment in neuronal proliferation [53] and its administration boosts widespread neurogenesis after an ischemic insult [54]. The GM may also exert region-specific effects in the brain. GF mice upregulate genes involved in myelination of the prefrontal cortex, but not in other regions, which can be reversed by colonization with commensal bacteria [55]. The microbiota also affects glial development and function. Microglia participate to several events during growth, such as synaptic pruning, promotion of network wiring and release of cytokines necessary for neuronal differentiation. Defects in microglia occur in GF conditions, as they become dysfunctional due to disruption of developmental genes and immune response pathways [56]. SCFA-producing bacteria may mediate such an effect, as a supply of butyrate restores normal microglial morphology [57]. Astrocytes may also be regulated by gut microbes, as some bacterial species produce tryptophan-derived metabolites which bind to the aryl hydrocarbon receptor (AHR), present on astrocytes’ membrane, stimulating type I interferon signaling and reducing inflammation and disease scores in experimental autoimmune encephalomyelitis (EAE) mice, a model of multiple sclerosis (MS) [58]. 

Finally, the BBB integrity is influenced by the microbiota. Depletion of microbial community leads to a 75% loss of tight junction proteins occludin and claudin-5 in the BBB of mice, increasing the CNS susceptibility to exogenous stimuli such as lipopolysaccharide (LPS) and oxidative stress, both potent inducers of systemic and neuroinflammation [59]. One study demonstrated that this defect may be restored by the supplementation of propionate or butyrate, highlighting a potential role of SCFAs also in BBB homeostasis [60]. 

### 3.3. Diet and Microbiota on the Immune System: An Indirect Link to the CNS

Nutrition and microbiota also impact the immune system, as priming of the adaptive immune response towards an antigen may depend on metabolic factors. The heavy supply of FAs observed in mice fed on high-fat diets elicits a profound transcriptional change in CD4 T lymphocytes due to the activation of acetyl-CoA carboxylase 1, an important enzyme regulating FA metabolism in cells. This protein in turn switches on transcription factors involved in the differentiation towards Th17 cells [61]. Indeed, saturated FAs derived from diet directly enhance the differentiation and proliferation of Th1 and/or Th17 cells and their escape from the intestinal environment, which favors the rise in autoimmune events in the CNS [62]. Factors from protein catabolism also modify the immune response in the gut. Dietary serine [63] and methionine [64] affect the activation of naïve T cells and differentiation to a Th17 effector cell, respectively, by altering the epigenomic pattern of the cell. Indeed, dietary methionine restriction dampened neuroinflammation in a murine model of MS by decreasing Th17 cell activation, delaying disease onset and progression. In this context, it is not surprising that metabolic disorders are associated with a chronic low-grade Th1- and Th17-mediated inflammation [61], which has a strong pull towards the development of autoimmune diseases, including MS and chronic inflammatory demyelinating polyneuropathy (CIDP) [65,66]. Recently, Doneddu et al. reported that higher fish consumption may decrease the risk of CIDP [67], an association possibly mediated by the anti-inflammatory effects of ω-3 PUFAs, highly contained in seafood. 

Commensal bacteria are essential to maintaining gut barrier integrity and participate in proper development of both B and T lymphocytes. The microbiota is involved in B cell maturation and response, shaping its function towards a tolerant state during mucosal exposure while rapidly switching to attack mode in case of entry in the systemic circulation [68]. The colonization of microbiota is essential in triggering an immune response, as the recruitment of autoreactive B and T cells in CNS demyelinating lesions relies on the availability of target autoantigen and commensal bacteria [69]. Indeed, GF mice showed a reduced inflammation in the EAE model, as the immune system failed to mount a Th17-mediated response [70]. Nonetheless, gut bacteria may also modulate immune response by shaping the fate of T cell differentiation in the gut. *Bacteroides fragilis* stimulates differentiation towards T_reg_ cells [71]. Additionally, butyrate increases the expression of T_reg_ cells and reduces EAE symptoms and axonal damage, suppressing at the same time the activation of Th17 cells [62]. An important therapeutic mechanism of dimethyl fumarate, a first-line approved drug for MS, is the activation of hydroxycarboxylic acid receptor 2 (HCAR2), a transmembrane receptor binding β-hydroxybutyrate and butyrate, which induces phenotype switching in microglia from pro-inflammatory to a neuroprotective state [72,73]. 

## 4. Roles of Diet and Microbiota in Neurodegenerative Diseases

Metabolism, diet and microbiota represent the three main hubs of a complex network that participates in brain function and preservation. Disruption of this equilibrium may occur in any of these axes, causing perturbations in CNS homeostasis. Obesity, T2D, dyslipidemia and hepatic steatosis are key markers of metabolic syndrome and each affects the nervous system through shared and independent mechanisms, resulting in common downstream pathways such as altered energy homeostasis, oxidative stress, neuroinflammation and neuronal death. Dietary habits also have profound effects on neural function, and specific dietary behaviors are associated with neurodegenerative diseases, especially when found during midlife, a time period in which habits and lifestyle are assumed to be stable across the years, proving a potential biomarker for long-term exposure to investigated factors. Finally, the microbiome represents an attractive novel target for the development of neuroprotective interventions. Dysbiosis has been robustly associated with metabolic disorders [74,75] and affects several aspects of health, promoting endotoxemia, systemic inflammation and loss of beneficial microbial metabolites. Additionally, it produces a wide array of amyloid proteins with prion-like properties, potentially serving for cross-seeding and propagation of pathologic protein aggregates from the gut to the CNS. The complex network organization of the CNS in anatomically and functionally distinct neural circuits implicates variation in their biology and individual susceptibility to external stimuli. The hippocampus, substantia nigra and motor system (cortex and spinal cord) are the most extensively studied areas of the CNS, each presenting unique peculiarities in terms of energy metabolism, cellular architecture, and response to damage. Dissecting the myriad interactions occurring between these systems and the external/internal world may prove an effective strategy to develop new possibilities for the prevention and treatment of neurodegenerative diseases. 

### 4.1. Alzheimer’s Disease

AD is a neurodegenerative disorder characterized by the deposition of amyloid-β (Aβ) plaques and neurofibrillary tangles. Typical AD manifests as selective memory impairment, then progresses with loss of language, visuospatial abilities, and other cognitive functions up to total activity dependence. The main genetic risk associated to AD is the presence of an ε4 allele variant in the apolipoprotein-ε (*APOE4*) gene. ApoE is the main protein involved in cholesterol transport within the brain. Table 1 summarizes the key features associating metabolic disorders, nutrients and microbiota with AD.

#### 4.1.1. AD and Metabolic Disorders

The rise of metabolic disorders and AD prevalence in Western countries suggests that an intimate link exists between metabolism and dementia. Obesity during midlife is associated with an increased risk of dementia up to 74%, independently from hypertension, diabetes, *APOE* genotype and other cardiovascular risk factors [76,77,78]. In particular, central adiposity has a predominant effect, as measurements of waist circumference better associate with AD compared to body mass index (BMI) [79]. In animal models of AD, worsening of memory impairment, brain atrophy and increased β-amyloid accumulation were observed in response to a high-fat diet [80,81]. Furthermore, glial cells may be particularly sensitive to diet, since an increase in astrocytosis and microglial activation occurs in AD mice after chronic exposure to a Western diet [82]. The relationship between AD and obesity holds true also in the opposite direction. Indeed, animal models of AD showed an increased susceptibility for increased adiposity and insulin resistance compared to wild-type (WT) mice following a high caloric diet [83,84].

Besides obesity, T2D is an important metabolic risk factor for dementia, leading some authors to define AD as a “type-3 diabetes”. Three meta-analyses provided strong evidence that T2D significantly predicts cognitive decline and AD, with up to 50% higher risk in the diabetic population compared to non-diabetics [85,86,87]. Besides peripheral insulin resistance, aberrancy in the brain insulin pathways occurs in diabetes and AD. Defects in the insulin/IGF-1 pathway are prevalent in the brain of both AD patients [88,89] and animal models [90]. Conversely, the cerebral amyloid production and tau phosphorylation significantly increase in diabetes without overt dementia [91,92]. A decreased insulin signal in neurons promotes mitochondrial dysfunction, increasing the susceptibility to oxidative stress, tau phosphorylation, neuroinflammation and altered neurotransmission [93]. Recent studies also highlighted the role of autophagy as a mechanistic link between insulin resistance and β-amyloid accumulation. High sugar consumption leads to hyperactivity of mammalian target of rapamycin (mTOR) in neurons, which in turn inhibits autophagy [94]. Subsequently, the accumulation of undegraded autophagosomes contributes to abnormal amyloid precursor protein (APP) processing, leading to enhanced Aβ generation [95]. 

Dyslipidemia increases the risk of AD. High total and low-density-lipoprotein cholesterol (LDL-C) levels during midlife were most consistently associated with chances of future dementia [96]. A recent study reported that early-onset AD patients have high LDL-C levels and 3% of them harbor rare variants in the *APOB* gene [97], providing both epidemiological and biological links between aberrant cholesterol metabolism and AD. Furthermore, hypercholesterolemia associated with a heavier burden of AD pathology and cerebral amyloid angiopathy in autoptic cases [98], while animal models of AD showed worsening of Aβ accumulation and cognitive deficits upon diet-induced hypercholesterolemia [99]. Thus, targeting lipid profile may be useful for AD prevention. In this context, observational studies showed a protective effect of statin exposure on risk of dementia but randomized clinical trials evaluating the effects of lipid-lowering drugs on AD yielded non-significant results [96]. Finally, recent evidence suggests that MAFLD is associated with reduced cognitive function and lower brain volume in adults [100,101]. Epidemiological findings are further supported by experimental evidence, as stimulation of MAFLD induced AD-like pathology in WT mice and worsens the phenotype in AD mice models [102]. 

Supporting the bi-directional link between metabolism and neurodegeneration, the *APOE4* genotype may exert an important role as a disease modifier in relation to obesity and T2D with AD. A diet-induced weight gain exhibited a stronger negative impact in AD mice carrying the *ApoE4* allele, compared to AD mice with an *ApoE3* allele [103]. Conversely, a higher incidence of metabolic disturbances is present among human *APOE4* carriers, including obesity, hypercholesterolemia and T2D [104,105]. 

#### 4.1.2. AD and Diet

Most studies evaluating the role of diet in AD focused the analysis of isolated nutrients or food. Both quantity and quality of dietary fat matters for AD susceptibility. A positive relationship between a high consumption of saturated and trans-unsaturated fat and AD risk was demonstrated in several large observational cohorts [106,107,108]. In one study with an average follow-up of 21 years, intake of saturated fat at midlife was linked to increased incidence of dementia and AD, especially among carriers of the *APOE4* allele [109]. 

A deficiency in ω-3 PUFAs is present in the brain of AD patients [110]. A negative association was also found between DHA plasma levels and burden of cerebral amyloidosis [111]. Administration of DHA in aged rats alters membrane composition and gene expression in the brain, including an increase in transthyretin transcription, an Aβ protein scavenger, in the hippocampus [112,113]. The effects on gene expression may be regulated by epigenetic modifications, as DHA was shown to inhibit histone deacetylation and demethylation in neuroblastoma cells [114]. DHA properties on neuronal membranes are also important for proper APP processing towards the non-amyloidogenic pathway, as it decreases the activity of β- and γ-secretase cleaving enzymes [115]. A systematic review on the role of ω-3 FAs supplementation in AD animal models concluded that a long-term administration is associated with a reduction in Aβ plaques and neuronal loss, and improvement in cognitive function [116]. DHA may also exert its anti-amyloidogenic effect through indirect mechanisms, as it modulates neuroinflammation [115] and protects from endothelial vascular damage [117]. In humans, intake of DHA through either supplementation or fish consumption was found protective against AD in observational studies [118,119,120], although a lower response may be found in *APOE4* carriers [121,122]. Notably, the association between DHA and AD was not confirmed in randomized clinical trials [123,124]. Such discrepancies may be due to differences in study length and design between observational and interventional studies and suggest that a benefit for ω-3 PUFAs would be most evident when taken for a preventive rather than interventional purpose [125]. Furthermore, one recent study showed that a high ω-3 supplementation resulted in just a 28% increase in CSF DHA concentrations, despite a 200% increase in plasma levels [126], suggesting that drawbacks in drug dosage and delivery may have accounted for the failing results observed in clinical trials.

Oxidative stress is an important mechanism contributing to AD. Chronic hyperglycemia induced by high sugar intake exerts an important pressure on the mitochondrial machinery, promoting an excessive production of reactive oxygen species (ROS). Compared to glucose, fructose is as much as ten times more reactive, providing a potential explanation for the cognitive impairment observed in mice fed with a high-fructose diet [127]. Peroxidation of lipid precursors alters neuronal membrane composition, which may enhance abnormal APP processing [128]. Oxidative lesions in the DNA may also interfere with substrate recognition of DNA methyltransferases, leading to a state of global hypomethylation, which enhances the expression of the APP protein and Aβ deposition [129]. However, although the use of antioxidants, such as vitamin C and E, in AD may be attractive, results from human studies are conflicting. Long-term intake of vitamin E was the most consistent dietary antioxidant associated with reduced AD risk, while vitamin C, beta-carotene and flavonoids were not significant [130]. Others did not find any association between intake of vitamin antioxidants and AD [131,132]. Randomized trials also failed to demonstrate significant protection [133,134]. Still, a recent large prospective study highlighted a protective role of dietary flavonols, a class of polyphenols with antioxidant properties, on AD risk [135]. Like ω-3 PUFAs, the long-term exposure to antioxidants may be effective in aiding protection compared to the short-term administration employed in clinical trials. Coffee exerts beneficial effects on short-term memory and cognition. Pre-clinical studies suggested neuroprotective effects in models of dementia [136]. However, a robust meta-analysis of prospective studies found no association between coffee and AD [137].

A substantial body of evidence showed that specific dietary patterns may exert a protective role against AD. Long-term adherence to MeD is associated with a substantial risk reduction, independently from environmental, genetic and cardiovascular factors [30,138,139]. Indeed, MeD positively correlates with scores of learning and memory function tests, as well as volumes of dentate gyri [140]. One study also reported a reduction in AD mortality in patients consuming a MeD [141], but this finding still seeks replication by others. Caloric restriction has been shown to protect against AD in humans [108] as well as in animal models it delays AD onset and progression, decreasing Aβ deposition and enhancing neurogenesis [142]. No human studies properly evaluated the role of Western diet in AD risk, but experimental models consistently proved detrimental effects of diets high in fats and sugars on neurodegeneration [117]. 

#### 4.1.3. AD and Microbiota

The microbiota is altered in AD. Small, observational human studies revealed that AD patients show reduced community diversity, with a relative increased abundance of *Bacteroides* phylum and a decrease in *Firmicutes* compared to age- and sex-matched healthy controls [143,144]. In such conditions, opportunistic Gram-negative bacteria such as *Escherichia coli, Shigella, Helicobacter* and *Odoribacter* arise, favoring an inflammatory milieu in the gut and blood circulation, with higher levels of tumor necrosis factor (TNF)-α, interleukin (IL)-1β, IL-6 and nucleotide-binding oligomerization domain, leucine rich repeat and pyrin domain containing 3 (NLRP3) [145]. Meanwhile, beneficial commensals like *Bifidobacterium* and SCFA-producing bacteria, such as *Faecalibacterium prausnitzii, Eubacterium and Roseburia*, are reduced, suggesting an inadequate support of microbial metabolites to the brain [144,145]. Animal models of AD also display important alterations in the GM, although a consensus among studies is lacking [146,147,148,149]. Interestingly, Honarpisheh et al. reported that changes in the microbiota precede the deposition of cerebral Aβ in an AD model and were accompanied by an impaired intestinal epithelial barrier (IEB), Aβ deposition along gut vessels and an early systemic inflammation which disappeared after symptom onset, suggesting the existence of a pre-symptomatic phase where dysbiosis and peripheral inflammation may trigger Aβ aggregation in the CNS [150]. 

Manipulations of the microbiota also revealed its contribution in AD. Depletion of the microbial community by either the GF approach or broad-spectrum antibiotics alleviated cognitive impairment, Aβ plaque burden, glial reactivity and neuroinflammation [151,152]. Furthermore, colonization of GF AD mice with microbiota from WT mice led to higher cerebral Aβ, an effect more pronounced when GF mice were colonized with microbiota from transgenic animals [151]. Fecal microbial transplantation (FMT) from AD patients worsened the severity of the disease and increased levels of the NLRP3 in transgenic mice, while transplantation in WT mice did not elicit cognitive deficits but raised levels of intestinal NLRP3, supporting the hypothesis that intestinal inflammation is an active player, although not the driver, in disease pathogenesis [153]. Nonetheless, FMT from WT to AD mice alleviated clinicopathological markers of AD, intestinal inflammation and IEB integrity by restoring normal microbiota and SCFA production [146,154]. 

Despite the effects of dysbiosis in AD, one study reported that murine microbial composition was strongly associated with the *APOE* genotype, independently from sex and disease status [155]. The *APOE4* allele was linked to higher levels of *Erysipelotrichaceae*, a family including pro-inflammatory bacteria, while the protective *APOE2* allele was positively correlated with family *Ruminococcaceae*, rich in SCFA-producing species. Thus, not may only the microbiota affect AD onset and progression, but also AD-related genetic factors may shape microbial composition and changes during lifespan.

The microbiota may affect AD-related neurodegeneration through several mechanisms. The increased abundance in inflammatory pathogens in the gut lumen may favor the release of bacterial endotoxins, such as LPS, leading to the disruption of IEB integrity and intestinal inflammation. A landmark study reported greater levels of LPS and other Gram-negative bacterial molecules in the AD human brains compared to controls, which colocalized with Aβ deposition in plaques and vessels, suggesting potential interactions between LPS and Aβ [156]. Indeed, LPS enhances Aβ fibrillization in vitro [157] and potently induces nuclear factor kappa-light-chain-enhancer of activated B cells (NF-kB) signaling in neuronal cells, triggering inflammation [158]. Furthermore, LPS may affect Aβ transport in the circulation, increasing influx and decreasing efflux of Aβ across the BBB [159]. Chronic systemic injections of LPS for up to a week elicits cognitive dysfunction, hippocampal Aβ production and microglial activation in WT mice, suggesting that peripheral stimuli may trigger or exacerbate neurodegenerative processes [160]. Interestingly, LPS may exert prolonged effects, as Aβ deposition [161] and microglial activation [162] may persist up to a month after the suspension of the endotoxin infusion. 

Another recent hypothesis is that sources of Aβ may be present in the gut, potentially triggering or enhancing Aβ aggregation in the brain. Several bacterial species produce amyloid peptides, which serve as scaffolds for structural integrity and biofilm production. Such molecules share many aggregate-like properties with cerebral Aβ fragments and are recognized by toll-like receptor 2 (TLR2) on inflammatory cells, stimulating gut inflammation [163]. It could be possible that in a context of dysbiosis and impaired IEB/BBB, amyloid peptides may translocate in the circulation and enhance brain Aβ aggregation. For example, the *E. coli* producing amyloid protein Curli cross-seeds with α-synuclein and stimulates protein aggregation in both the gut and the brain in WT mice [164]. Furthermore, Curli showed amyloid-accelerating properties in the murine experimental AA amyloidosis (secondary amyloidosis) [165]. However, studies directly assessing the role of exogenous amyloid peptides in Aβ are missing. 

An alternative hypothesis may be that endogenous Aβ production takes place in the gut and subsequently spreads to the CNS. Indeed, higher Aβ levels were detected in the intestinal mucosa of both AD animal models [147] and patients [150]. Aβ expression increases in response to a high-fat diet in WT mice [166], while APP regulates the phenotype of both adipocytes and peripheral macrophages [167], suggesting a role of APP and its degradation products in lipid metabolism and modulation of the immune system. Nonetheless, studies failed to demonstrate a cross-seeding effect of oral and intravenous Aβ exposure on cerebral amyloidosis [168]. Still, intraperitoneal injections of Aβ-enriched extracts induced cerebral amyloid pathology 7 months after [169], suggesting that prolonged incubation times may be needed to appreciate the effects of peripheral stimuli to Aβ deposition. In such a scenario, peripheral macrophages have been proposed as candidate vectors for the transport of exogenous Aβ in the CNS [170]. Alternatively, axonal retrograde transport across peripheral nerves may be another route of Aβ dissemination. Amyloid deposition in the vagus nerve and celiac ganglions has been reported in patients with systemic amyloidosis [171]. Recently, Sun et al. showed that intra-GI injection of Aβ oligomers induced vagal and cerebral amyloidosis after 12 months [172], suggesting that neural transport may be considered as a potential route of spread of Aβ pathology towards the CNS.

Restoring normal SCFA levels may be another approach to target Aβ and neurodegeneration. Levels of SCFAs are reduced in AD animal models [173]. Indeed, SCFAs are able to inhibit Aβ aggregation in vitro [174]. Furthermore, sodium butyrate curbed high cholesterol-induced neuronal amyloidosis in neuroblastoma cell lines by stimulating antioxidant pathways and inhibiting amyloidogenesis [175]. SCFAs also affect microglial function by reducing the expression of inflammatory markers and phagocytic activity in vitro upon stimulation by endotoxins [176].

Considering the wealth of evidence about dysbiosis in AD, it seems feasible to explore whether interventions aimed at microbiota may affect disease pathogenesis. Several studies showed that probiotics based on *Bifidobacterium*, *Lactobacillus* or other beneficial commensals alleviated memory deficits, decreased the Aβ plaque burden and reduced neuroinflammation in AD animal models [177,178,179]. These effects may be mediated by boosting protein degradation machineries, such as the ubiquitin-proteasome system and autophagy, gut hormones’ release and SCFA production [180]. Furthermore, restoring normal microbiota allowed the recovery of intestinal inflammation [181] and alterations in glucose homeostasis [182], suggesting that interventions aimed at eubiosis may target both metabolic and neurodegenerative processes. Up to now, three clinical trials investigated the effects of a 12-weeks course of probiotics consisting of *Lactobacillus* and *Bifidobacterium* strains [183]. Unfortunately, they were unable to demonstrate strong efficacy on cognitive function, although they may improve markers of lipid profile and insulin resistance. 

### 4.2. Parkinson’s Disease

PD is the second most common neurodegenerative disorder among the elderly population, characterized by a loss of movement control, resulting in bradykinesia, muscle rigidity, tremor, gait difficulty and postural instability. Non-motor symptoms are also common, such as depression, anxiety, constipation and rapid eye movement sleep behaviour disorder (RBD), which may be apparent up to 20 years before the onset of motor features. Pathogenesis grounds on the loss of dopaminergic neurons in the substantia nigra and pathologic accumulation of α-synuclein (α-syn) into protein aggregates known to show off as Lewy body pathology. About 15% of PD patients have a positive family history, with mutations in *LRRK2* (LRRK2), *PARK7* (DJ-1), *PINK1*, *PRKN* (Parkin), and *SNCA* (α-synuclein) genes being the most common. Over the past two decades, a robust body of evidence has shed the light on the impact of dietary habits, metabolism and microbiota on PD incidence, revealing novel mechanisms contributing to dopaminergic neurotoxicity (Table 2).

#### 4.2.1. PD and Metabolic Disorders

No relationship has been found between BMI and PD in most epidemiological studies [184,185,186,187]. Only one cohort in Finland reported a positive association between BMI and PD, revealing an independent risk in obese (BMI > 30) compared to non-obese individuals [188]. In addition, other markers of obesity such as higher triceps skinfold thickness [184] and waist-to-hip ratio [185] show a greater PD risk, suggesting that central adiposity may be a better indicator of disease risk compared to overall body mass. 

Prospective cohort studies suggest that T2D positively associates with PD, reporting about 35-40% higher risk in the diabetic population [189,190,191]. Nonetheless, no association was reported in two large US cohorts [192] and in the Cancer Prevention Study Cohort [186], leaving the issue still undefined. It is possible that confounding or intermediate factors may play a role as risk modifiers. For example, hyperuricemia is a known risk factor for T2D [193], but at the same time is protective against PD (see below). Indeed, other clues suggest that the two conditions are intimately related. PD patients with diabetes show worse motor symptoms, such as postural instability and gait difficulties, compared to non-diabetic cases [194]. Experimental studies supported the hypothesis that insulin resistance in dopaminergic neurons is an important contributor to PD-related neurodegeneration, by decreasing survival signals and enhancing mitochondrial dysfunction, oxidative stress and local inflammation [195]. A reduced activity of peroxisome proliferator activated receptor gamma coactivator 1-α (PGC1α), a transcriptional regulator mediating mitochondrial biogenesis, is found in both diseases, suggesting shared mechanisms of damage [196]. Finally, two studies showed that the use of anti-diabetic drugs such as metformin and dipeptidyl peptidase-4 inhibitors may reduce the incidence of PD in diabetic patients [197,198], raising the possibility that therapeutic interventions for metabolic disorders may have an impact on the prevention of future neurodegenerative diseases.

The relationship between hypercholesterolemia and PD is uncertain [199]. The most recent meta-analysis, collecting 21 studies of which there were 8 prospective cohorts with a total of 11,188 incident PD patients among almost 1 million individuals, suggested that high serum total cholesterol and LDL-C were protective against PD (RR = 0.76 and RR = 0.86, respectively) [200]. Higher cholesterol levels in early PD were linked to slower disease progression [201] and lower iron deposition in the substantia nigra of PD patients [202], highlighting a potential role of cholesterol metabolism in dopaminergic neurotoxicity. However, results from experimental studies diverge from epidemiological evidence. Indeed, the induction of serum hypercholesterolemia worsened the phenotype in a PD animal model [203], while in WT mice it led to dopaminergic loss and motor behavioral abnormalities reminiscent of PD [204]. Thus, further research is needed to clarify the role of cholesterol in PD.

Whether a link exists between MAFLD and PD is currently unknown. Although a positive association exists for T2D with both MAFLD and PD, a transitive property has not been shown yet [205]. Conversely, the only study investigating the prevalence of MAFLD in PD patients reported lower rates of hepatic steatosis compared to matched controls [206]. For instance, DJ-1, a protein involved in oxidative stress and inflammatory response, seems a converging point between the two diseases as genetic mutations cause autosomal recessive forms of PD [207], while an altered protein function has been associated with MAFLD progression towards fibrosis and cirrhosis [208]. Treatment of MAFLD is of paramount importance, as cirrhosis exerts detrimental effects on dopaminergic neurons, such that up to 20% of cirrhotic patients may develop chronic parkinsonism [209]. 

#### 4.2.2. PD and Diet

The diet–PD relationship shows strong positive or negative associations with specific foods, a feature still not observed in other neurodegenerative diseases. Milk intake is the most consistent dietary factor associated to an increased risk of PD across multiple studies, with a relative risk (RR) of 1.6 when comparing higher vs lowest category of intake [210,211,212]. Such a relationship is not explained by levels of vitamin D, calcium and total amount of diary fats, or its saturated/unsaturated ratio, suggesting that other factors are implicated. A lower risk of PD was observed in coffee drinkers compared to non-drinkers [213,214].

Many studies revealed that this association is mainly driven by caffeine consumption and appears to benefit males more than females. Sex hormones may be important modifiers of caffeine’s protective role on PD, as hormone replacement therapy in postmenopausal women may counteract coffee’s beneficial effects when compared to postmenopausal women who did not take hormone replacement therapy [215]. Caffeine may exert its neuroprotective role by acting as an adenosine A2a receptor antagonist [216]. Blockage of this receptor may inhibit glutamate excitotoxicity, increasing the chances of neuronal survival. Furthermore, caffeine downregulates NO production, inflammatory cytokines and microglial activation [217]. Chronic low doses have been evaluated in clinical trials, witnessing partial, symptomatic relief of bradykinesia, rigidity, freezing and depression [218,219]. Such findings gave new hope to investigate the potential of more selective A2a receptor antagonists, such as istradefylline, as a therapeutic adjunct in PD management, with clinical trials demonstrating a modest amelioration of PD symptoms and levodopa-induced dyskinesias in treated patients [220,221,222]. 

Uric acid, the end-product of purine metabolism, is a potent antioxidant contained in several foods, especially meat, fructose and alcoholic beverages. A substantial body of evidence demonstrated that urate levels are inversely related to PD, with a 20 to 40% risk reduction [223]. Both genetic and environmental factors affecting urate concentrations may act as modifiers on PD risk. Polymorphisms in the *SLC2A9*, a urate transporter, is the strongest genetic determinant of its body concentrations and variants linked to increased urate levels appeared to postpone onset of PD symptoms in carriers [224]. Besides its effects on disease risk, uric acid may also influence disease course. Indeed, detection of higher urate levels in the early phases of PD predicts a slower progression, an effect more pronounced in men compared to women [225]. Urate protects dopaminergic neurons from death probably via the activation of the antioxidant response pathway, as observed in experimental models of PD [226]. Considered the overall evidence linking uric acid and PD, several authors struggled to develop urate-related compounds for use in clinical trials (e.g., NCT02642393, unpublished data), although results have not been successful so far.

Differently from AD, macronutrients do not seem to play a prominent role in PD. The relationship with fat intake is uncertain, as either positive, negative or no association were found [217]. Only one study reported an increased risk with saturated fatty acids [227], while ω-3 PUFA appeared protective in two prospective cohorts [189,228], but not others [228,229]. Nonetheless, animal studies showed that a high-fat diet exacerbates parkinsonian symptoms and neuronal death in substantia nigra and striatum [217]. Moreover, short-term administration of DHA delayed the onset and improved the severity of levodopa-induced dyskinesias in parkinsonian primates [230]. Protein intake failed to associate with any risk of PD [231], but a low-protein diet may be helpful in PD by favoring absorption of levodopa in treated patients, allowing a reduction in daily drug dosage of up to 40% [232,233]. Finally, studies addressing a direct role of carbohydrate intake in PD, independently from diabetes, failed to reveal any significant association [234].

Dietary patterns may offer a synergic protection towards PD due to enrichment in antioxidants and beneficial factors for neuronal function. In two large prospective cohorts, the use of a prudent diet, made by a high intake of fruits, vegetables and fish, showed a RR of 0.78 [235]. The MeD may also offer protection against PD [191,236], but more longitudinal studies are needed to corroborate these findings. Like AD, caloric restriction appears protective against risk of PD in humans and animal models [33]. 

#### 4.2.3. PD and Microbiota

**Figure 2:**
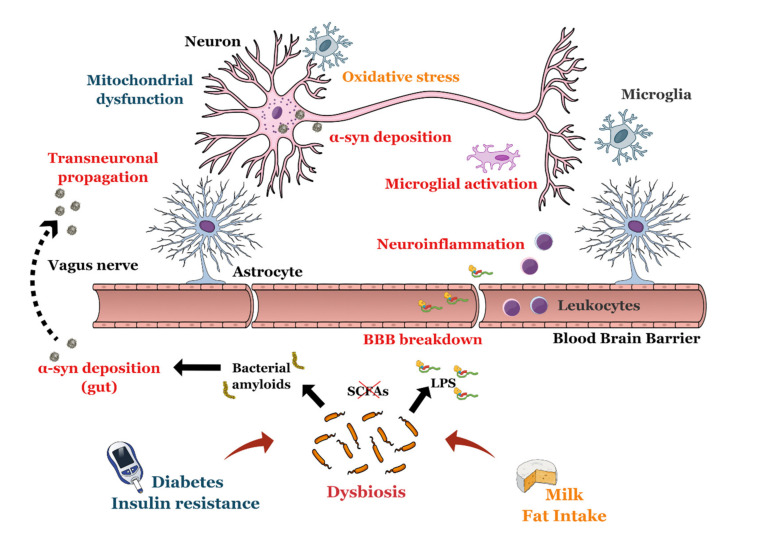
Mechanisms of PD neurodegeneration. Dysbiosis is a prominent feature of PD, which may be further influenced by diabetes and fat intake. The altered GM induces numerous alterations such as endotoxemia, reduced mucin thickness and SCFA levels. Endotoxemia disrupts BBB, increases α-synuclein deposition, stimulates microglial activation and neuroinflammation. At the same time, diabetes and fat/milk intake may exert independent effects such as mitochondrial dysfunction and oxidative stress.

Robust evidence supports a role for dysbiosis in PD-related neurodegeneration (Figure 2). Around 20 studies have been published since 2015, highlighting the presence of specific microbial signatures associated to the disease. Two meta-analyses showed that higher abundances of families Verrucomicrobiaceae and Lactobacillaceae, and genera Akkermansia, Lactobacillus, Bifidobacterium were found in PD patients compared to controls, together with lower abundances of SCFA-producing bacteria, including families Lachnospiraceae and Prevotellaceae and genera Faecalibacterium, Roseburia, Blautia and Prevotella [237,238]. Some studies also reported higher levels of pro-inflammatory bacteria such as E. coli/Shigella and Ralstonia. Recently, Wallen et al. published data on the largest cohort study of GM in PD, revealing that three clusters of differentially abundant bacteria may be found in PD: 1) higher levels of opportunistic, LPS-secreting pathogens, 2) reduction in SCFA-producing bacteria, and 3) higher abundance of carbohydrate-metabolizing bacteria, such as Lactobacillus and Bifidobacterium [239]. Alterations in the gut microbiome may also be observed years before motor symptoms appear, as patients with RBD, common in the prodrome phase of PD, display similar GM patterns [240]. The potential neurotoxic effects of dysbiosis may span long time periods, as a longitudinal 2 year follow-up demonstrated the stability of the GM alterations [241]. Correlations with clinical phenotype and severity have been reported [238], suggesting that the microbiota may play a prognostic role. PD medications may also affect GM composition, as specific signatures have been related to levodopa and entacapone use [242]. Conversely, microbiota may alter levodopa bioavailability, as the abundance of Enterococcus and Lactobacillus species stimulates levodopa degradation pathways in the gut, potentially contributing to higher drug dosage and related complications in clinical practice [243]. 

Both environmentally [244] and genetically [245] induced animal models display altered GM. Depletion of microbiota induced by GF condition or chronic broad-spectrum antibiotics was protective against dopaminergic loss and motor dysfunction in PD mice [246,247]. In addition, colonization of α-Syn-overexpressing mice with microbiota of PD patients worsened motor deficits [247] while FMT from WT mice ameliorated clinical impairment in a toxin-induced PD model [248]. All these findings support an active role of microbiota in PD pathogenesis. 

The PD-related GM is associated to important functional changes in the gut lumen affecting numerous metabolic pathways, such as amino acid, carbohydrate, xenobiotic and β-glucuronate metabolism [249,250]. Higher levels of *Akkermansia municiphila* stimulate mucin degradation, increasing host susceptibility to IEB disruption. Furthermore, *A. municiphila* and *Bilophila wadsworthia* affect sulfur metabolism in PD leading to higher secretion of sulfite, a known neurotoxin causing mitochondrial dysfunction [251]. In addition, levels of taurine-conjugated bile acids, exploited in the gut lumen as a source of sulfite production, positively associated with scores of clinical severity. A greater endotoxin exposure was also observed in PD patients compared to healthy controls [252], which was accompanied by increased markers of intestinal permeability [253] and gut inflammation [254]. Interestingly, epidemiological data suggest that patients with inflammatory bowel disease carry a higher risk of getting PD [255], with genetic variants in *LRRK2* conferring a shared risk of Crohn’s disease and PD in the general population [256]. Further evidence of a link between intestinal inflammation and dopaminergic neurotoxicity was recently reported by Kishimoto et al., who showed that a chronic mild gut inflammation exacerbates neuropathology and motor deficits in α-Syn-mutant mice [257]. The higher endotoxin load allows evasion of immune defences in the gut and migration towards distant sites, potentially causing toxic effects in the CNS. Intrastriatal injection of LPS has been shown to replicate PD-like phenotype and progressive neurodegeneration in mice [258]. Studies on animal models demonstrated that systemic injection of LPS elicited a marked neuroinflammatory response in the Substantia Nigra of WT mice, leading to a progressive, selective loss of dopaminergic neurons, persisting months after cessation of exposure [259,260]. Activation of TLR4 on immune and glial cells is an essential step in LPS-mediated neurodegeneration, as TLR4-knockout PD mice show a reduction in intestinal and brain inflammation, motor deficits and neurodegeneration [261]. The higher susceptibility of the substantia nigra may be related to microglial activation, as this region exhibits a 4.5-fold higher density of microglial cells compared to hippocampus [262]. Regional differences in LPS-induced BBB disruption has also been observed, with the mesencephalon showing the highest vulnerability [263]. 

Another important mechanism of gut–brain axis dysfunction in PD is related to the aggregation and spread of pathologic α-syn into the CNS of PD patients. More than 30 years ago, Wakabashi et al. first detected the presence of Lewy bodies in the submucosal and myenteric plexuses across the GI tract of PD patients [264]. Subsequent studies highlighted that pathologic α-syn aggregation in the gut occurs in 65–85% of disease subjects [265,266], distributing with a rostro-caudal gradient of decreasing frequency, with the highest burden observed in lower oesophagus and stomach. Furthermore, pathological α-syn may be detected in prodromal stages of PD, preceding diagnosis of 10-20 years [267,268], and its burden positively correlated with the severity of constipation and extent of enteric neuronal loss in PD cases [269]. Pathologic α-syn staining also matched with grading of *E. coli* staining and endotoxin exposure in colonic biopsies of PD subjects [253], which advocates for a dysbiosis-induced mechanism of α-syn aggregation in the ENS. Recent evidence unveiled non-neuronal functions of physiological α-syn in the gut, such as antimicrobial and chemoattract activities [270]. Indeed, α-syn aggregates upon LPS binding and elicits a potent inflammatory response in the gut and brain by specifically activating TLR4/NLRP3 inflammasome pathway [271,272]. Furthermore, recent studies demonstrated an increased aggregation capacity of α-syn upon oral exposure to Curli which accelerated PD pathology and disease onset in mice overexpressing human α-syn [273]. Interestingly, enteroendocrine cells, which lie on the mucosal lining in direct contact with the gut lumen, have been proposed as a candidate site for nucleation of α-syn aggregation and transmission to the ENS thanks to its neuronal-like properties [274].

Braak et al. proposed a pathogenic model for PD in which the potential spread of an unknown neurotropic pathogen from the gut to the CNS triggers progressive neurodegeneration first in the dorsal motor nucleus of the vagus (DMNV) nerve which then propagates to the substantia nigra and other brain regions via trans-neuronal axonal routes, sustained by the prion-like properties of pathologic α-syn [275]. Supporting Braak’s view, active gut-to-brain neural transport of α-syn aggregates along the vagus nerve has been demonstrated in mice experimentally injected with α-syn preformed fibrils in the intestinal wall, which caused delayed, progressive PD-like neuropathology and motor impairment [276,277]. Epidemiological studies suggested that PD risk may be lower in individuals who underwent truncal vagotomy in the preceding 10-20 years [278], although findings are not always concordant. In addition, alternative routes may be exploited by pathologic α-syn to reach the CNS, such as the para-/sympathetic nerve pathways relaying on the neurons in the intermediolateral column of the spinal cord [279]. Interestingly, α-syn assemblies injected systemically were able to cross the BBB and distribute in the CNS [280], suggesting that blood transport may offer an additional way to reach and damage the substantia nigra. 

Despite early promising findings, a study assessing the diagnostic utility of α-syn pathology in colonic mucosa failed to show specificity, since control subjects also display aggregated α-syn staining in the ENS [281]. Studies on mice also showed that α-syn deposition occurs physiologically with aging [282], suggesting that this is a common age-related phenomenon upon which environmental factors, such as dysbiosis, toxin exposure and others, may trigger self-renewable protein aggregation and spread to the brain, causing neurodegeneration. 

SCFAs may also play a role in PD-related pathogenesis. Decreased fecal concentrations of SCFAs were detected in PD patients compared to healthy controls [283], in line with the observed reduction in SCFA-producing bacteria. Experimental studies further showed that oral administration of sodium butyrate attenuates behavioral and pathologic alterations in PD mice through amelioration of IEB integrity, inflammatory response and boosting neuronal autophagy [284,285]. However, other studies revealed that SCFAs may be deleterious in PD as they exacerbate PD phenotype in GF mice overexpressing α-syn [247].

Supporting the potential application of a GM-based therapy, preclinical studies showed that probiotics and prebiotics may offer protection against PD [286]. A recent trial demonstrated that fermented milk containing a mixture of probiotic strains and prebiotic fibers improved constipation in patients with PD compared to control milk [287]. Furthermore, a 12-week intervention with a probiotic mixture resulted in positive clinical and metabolic outcomes in treated PD patients compared to placebo [288]. Interestingly, robust evidence supports a role for probiotics in the treatment of major depressive disorders, showing potential for translation of these results also in PD-related depression [289]. Nonetheless, it is important to mention that most commercially available probiotics contain *Lactobacillus* strains, a genus found abundant in the gut of PD cases. Thus, a careful and detailed assessment of the role of specific bacteria in disease pathogenesis is warranted to ensure safe and efficacious microbiota-based formulas in the treatment of PD. In this regard, Goya et al. elegantly described a protective role of probiotic *Bacillus subtilis* against α-syn aggregation in a *Caenorhabditis elegans* model of synucleinopathy [290], demonstrating strong biological links between supplementation of this bacterium and reduction in neuropathology. 

### 4.3. Amyotrophic Lateral Sclerosis

ALS is a neurodegenerative disorder characterized by progressive loss of motor neurons in the brain and spinal cord, leading to generalized paralysis, eating difficulties and respiratory failure. Deposition of phosphorylated transactive response DNA-binding protein 43 (pTDP-43) protein aggregates in motor neurons and associated glia is a hallmark sign of ALS. Genetics exert an important role in disease susceptibility and alterations in chromosome 9 open reading frame 72 (*C9orf72*), superoxide dismutase 1 (*SOD1*), TAR DNA binding protein-43 (*TARDBP*), and fused in sarcoma (*FUS*), the most common ALS-related genes, show a high selectivity for motor neuron degeneration, although they have also been associated to other disorders, such as frontotemporal dementia and PD [291]. Applying mutations in these genes led to the development of ALS models, with the SOD1^G93A^ mouse model being the most commonly used. Still, exogenous factors may trigger or hasten motoneuronal damage. The peculiarity of ALS degeneration turned the attention to environmental exposure which may exert high stress on motor neurons, such as physical activity, trauma and smoking [292,293,294,295]. The relationship between metabolism, diet and microbiota received proper attention only in the last few years, but evolution in this new field may bridge some of the unresolved gaps in disease pathogenesis (Table 3). 

#### 4.3.1. ALS and Metabolic Disorders

A tight relationship exists between altered metabolism and motor neuron degeneration, with several studies highlighting a protective role for increased body weight and fat in ALS. Individuals who are overweight or obese show a 24% and 27% reduction in the risk of getting ALS, respectively [296]. Several studies also highlighted a prognostic role for body mass in ALS. Indeed, a lower BMI in the pre-symptomatic phase or at time of diagnosis predicts a shorter survival [297,298,299]. At the same time, ALS patients lose weight and body fat as the disease progresses [300], suggesting that the availability of fat stores is an important modulator of disease susceptibility and progression. Such association may be explained by hypermetabolism, defined as an increased resting energy expenditure despite adequate nutritional intake, which occurs in about 60% of ALS cases after the early phase [301], with higher rates observed in those with positive family history [302].

Studies in ALS mice suggested that skeletal muscles may be the primary origin of hypermetabolism [303]. A lipidomic study showed higher levels of glucosylceramide synthase (GCS), an enzyme synthesizing membrane lipids, in muscles of SOD1^G86R^ ALS mouse model as well as in a model of surgically-induced muscle denervation [304]. In turn, GCS was shown to increase the expression of genes involved in oxidative metabolism and assist motor recovery, suggesting that up-regulation of GCS may be a compensatory mechanism to favor muscle repair and re-innervation. Consistent with this hypothesis, partially denervated muscles increase glucose uptake and oxygen utilization at rest resulting in high lactate output [305]. Thus, hypermetabolism in ALS may represent an early mechanism by which muscles respond to denervation, which becomes maladaptive as motor neurons chronically fail to re-innervate the muscle tissue. Interestingly, a recent study showed that circulating levels of irisin, a myochine implicated in the regulation of body weight and metabolism, are increased in ALS patients with an hyper-metabolic status and correlate with the extent of functional and respiratory impairment [306].

Diabetes seems protective against ALS, although other factors may modify the interplay between the two disorders. In individuals from Western countries, the presence of diabetes in old age was protective against a future risk of ALS with reduction ranging from 40% to 70% [307,308].

Intriguingly, in the Asian population, diabetes was associated with an increased chance of getting ALS [309], suggesting that ethnic background may be an important modifier of gene-environment interactions. Reinforcing this concept, among Chinese ALS patients, the presence of diabetes was linked to a higher risk of mortality, showing a dose-dependent relationship with raising levels of hemoglobin A1c [310]. In contrast, a study comprising 1322 cases from the North American population failed to demonstrate an independent prognostic role of diabetes in ALS [311]. Following the above evidence, high blood glucose levels and serum markers of insulin resistance are less prevalent in ALS compared to controls and are inversely associated with survival [312,313]. Nonetheless, about a third of patients show impaired glucose tolerance and increased free FAs in the absence of overt diabetes [314]. Interestingly, mechanisms underlying hyperglycemia in ALS may be different from those observed in T2D. Levels of insulin and IGF-1 receptors are increased in skeletal muscles and CNS of patients [315,316], although these tissues show reduced glucose uptake [317,318]. Rather, mice overexpressing TDP-43 in skeletal muscles showed impaired Glut4 translocation on the outer membrane in response to insulin by upregulating Tbc1d1 [319]. Reduced levels of serum insulin and IGF-1 were seen in ALS [320], suggesting that impaired insulin secretion may be the culprit. Interestingly, a recent study reported loss of normal nuclear TDP-43 localization in islet cells of autopsied ALS cases, which was associated with the inhibition of early-phase insulin secretion in subsequent in vitro analyses [321]. All the above evidence supports the hypothesis that altered glucose homeostasis may be part of the multisystemic nature of ALS and may show off processes similar to those observed in motor neuron degeneration. 

**Figure 3:**
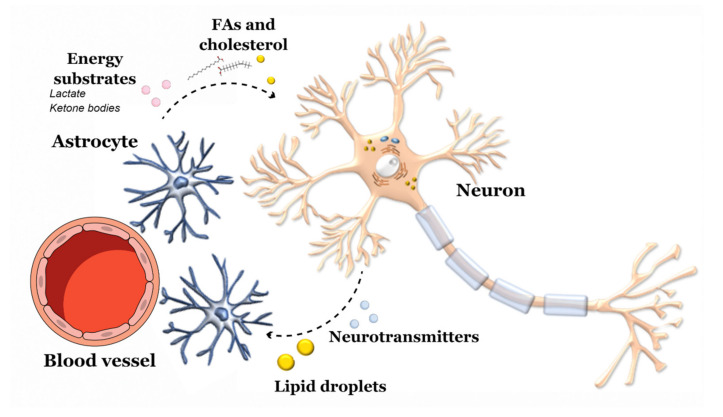
Neuron-glia unit in ALS. Neurons and astrocytes create a functional unit to regulate energy metabolism. Astrocytes uptake energy substrates (e.g., glucose, fatty acids) from the blood circulation and transfer them to neurons for ATP production. In stressful situations, neurons switch towards lipid metabolism. To this end, astrocytes supply alternative products such as lactate and ketone bodies, used by neurons for energy production and lipid droplet accumulation. Intraneuronal lipid droplets represent a defense mechanism against oxidative stress and protein aggregation, and their disposal requires transfer to astrocytes for degradation. Removal of extracellular neurotransmitters (e.g., glutamate) from synaptic clefts is important to maintain membrane potential and avoid excitotoxicity.

To date, epidemiological studies have failed to clarify the relationship between dyslipidemia and ALS (summarized in [312]). Only two studies prospectively assessed the role of lipid biomarkers in ALS incidence. The first one showed that high LDL-C and LDL-C/HDL-C ratio were associated with a higher risk of ALS, with the changing levels of lipid biomarkers occurring within 10 years preceding the diagnosis [312]. In the second study, the metabolomic profile of ALS patients showed broad alterations in lipid profile years before the disease onset, with plasma levels of diacylglycerides, triacylglycerides and urate being inversely associated with the disease, while cholesteryl esters, phosphatidylcholines and sphingomyelin showed positive associations with ALS [322]. Thus, dysregulation of lipid metabolism occurs in the early, prodromal phase of ALS and may be an integral feature of disease pathogenesis. Supporting this hypothesis, analysis from genome-wide association studies revealed that polygenic risk score for hyperlipidaemia was linked to a higher risk of ALS [323,324]. Nonetheless, a good prognostic role of elevated serum triglycerides and cholesterol was observed across several studies [325,326,327], suggesting that these alterations may represent a compensatory mechanism to the ongoing neuromuscular damage. Indeed, motor neurons from SOD1^G93A^ mice show defective bioenergetics from the embryonic stage onwards due to early mitochondrial dysfunction and activate a metabolic reprogramming favoring FA β-oxidation and ketone bodies formation over glucose catabolism [328]. Similarly, fast-fatigable muscle fibers of the SOD1^G86R^ mouse model, which are the earliest site of damage, undergo switch from glycolysis towards lipids use since the pre-symptomatic phase [329]. Besides their use as an alternative energy source, ketone bodies are exploited by neurons to produce intracellular lipid droplets, which have been shown to exert beneficial effects such as protection against oxidative stress and clearance of protein aggregates [330] (Figure 3). 

In such a context, the availability of fat stores may contribute to the modulation of disease progression, as witnessed by the positive correlation observed by subcutaneous fat content and survival in ALS patients [331]. Nonetheless, lipid droplet accumulation in neurons is detected only in the early phases of the disease [332], with a subsequent switch towards lipid catabolism potentially favoring ROS production and oxidative stress, exacerbating motor neuron degeneration. Besides the evidence from SOD1 models, post-natal deletion of TDP-43 downregulates Tbc1d1, a gene linked to obesity, leading to early body weight loss and death in mice, suggesting that TDP-43 may be critical for fat metabolism and early survival [333]. Finally, hepatic steatosis is a frequent finding in ALS [325,334], accompanied by ultrastructural abnormalities and mild liver dysfunction in patients [317]. Nonetheless, a pathogenic role of these findings has not been demonstrated so far. Considering the broad role of this organ in lipid handling and metabolism, further investigations are warranted.

#### 4.3.2. ALS and Diet

The rarity of the disease and the necessity of large and prolonged studies limit the proper evaluation of food exposures in ALS. Only few works are available to properly inform about the potential relationships between nutrients and disease risk. Current evidence does not support a role for most food nutrients in ALS [335,336], although exceptions have been highlighted for food-derived fat and antioxidants. 

How fat intake and ALS interact between each other is still a matter of debate. ALS patients had higher intake of total and saturated fat compared to controls during early stages of the disease, but conclusions are mostly based on case-control studies [337]. Similar to what observed for the association between lipid profile and ALS (see above), a diet enriched in fats was linked to longer survival in an observational study [338]. Preclinical studies also support the possibility that high fat diet interventions may extend lifespan in experimental animals [303]. Combining evidence from fat intake and hypermetabolism thus supports the possibility that high caloric nutrition may improve outcomes in ALS. Preliminary studies demonstrated the safety and feasibility of hypercaloric nutrition in ALS patients [339], while higher benefits may be obtained with a high-fat compared to a high-carbohydrate diet [340]. However, a recent randomized controlled trial failed to demonstrate a life-prolonging benefit in those receiving a high caloric-high fat diet compared to a standard diet [341]. 

Two large prospective cohorts assessed the role of ω-3 PUFAs in ALS. Fitzgerald et al. showed that a higher intake of PUFAs reduced the risk of ALS with a RR of 0.66 (95% CI, 0.53-0.81) [342]. Instead, the other study found not only that the majority of plasma PUFAs before diagnosis are not associated with ALS, but also that specific species, such as DHA, may paradoxically increase the risk [343]. Preclinical research also failed to clarify the role of ω-3 PUFA in ALS. A reduction in DHA content is observed in the spinal cord of ALS patients [344], which prompted the trial of DHA supplementation in ALS animal models. However, the results are discordant across studies, revealing both beneficial and detrimental effects. Indeed, although PUFAs may increase motor neuron survival by blocking inflammation and oxidative stress [345,346], they may at the same time stimulate lipid peroxidation and enhance protein aggregation in case of defects in DHA metabolism [347,348]. Collecting these findings, it may be possible that an increase in PUFAs may be an initial defense mechanism to counteract early cellular dysfunction by inducing anti-inflammatory and antioxidant response (possibly through lipid droplet formation), but with time, maladaptation occurs, favoring lipid peroxidation and a paradoxical increase in oxidative stress. Additional factors may be needed to stabilize the advantageous effects of PUFA intake. Addition of antioxidants such as tocopherol may confer protection through a synergistic action, as reported by both epidemiological and experimental studies [345,349]. Concerning protein and carbohydrate intake, studies failed to demonstrate robust association between these macronutrients and ALS [338,350]. 

Oxidative stress is one of the major mechanisms involved in motor neuron degeneration [351]. Consumption of food-derived antioxidants has been associated with a reduction in ALS risk, such as major carotenoids [352], vitamin E [353] and flavonoids [354]. Furthermore, results from a cross-sectional study suggested that a high intake of antioxidants, carotenes, fruits and vegetables was associated with better motor and respiratory functional scores [355]. Attempts to counteract oxidative stress showed survival benefits in ALS preclinical models, although these failed to be translated in humans [356]. Thus, despite oxidative stress participates in disease pathogenesis, counteracting this mechanism alone maybe enough to halt ALS progression. Similarly to PD, in ALS a possible preventive role has been suggested for coffee consumption [357]. To date, studies assessing the role of dietary patterns in ALS are scarce. Only one study investigated the role of MeD in ALS, but associations were mostly reported for some of its components and not for MeD as a whole [354]. Motor neurons heavily rely on the supply of nutrients to exert its functions and fail to adapt in response to dietary restriction. Indeed, SOD1-mutated mice exhibit worse functional outcomes and survival when exposed to caloric restriction [358]. 

#### 4.3.3. ALS and Microbiota

The research on the relationship between GM and ALS is at its infancy, but evidence for a pathogenic role is emerging. In human ALS, the microbiota shows decreased diversity and altered relative composition, although results were not uniform across studies, probably due to the small sample sizes [359]. To date, the largest study comparing 50 ALS patients with 50 controls reported an increased abundance of genera *E.coli-Shigella*, *Citrobacter*, *Akkermansia* and *Cyanobacteria*, together with lower levels of beneficial SCFA-producing bacteria such as *Bacteroides*, *Faecalibacterium*, *Eubacterium* and *Ruminococcus* [360]. Functional analysis revealed alterations in several pathways involving amino acid, nucleotide and carbohydrate metabolism [361]. The composition of the GM changes over time, mostly observed in genera belonging to the Bacteroidetes phylum [360]. 

ALS mice also show dysbiosis involving mainly SCFA-producing bacteria, *Akkermansia* and *Bacteroides* species, which precedes the onset of motor symptoms [362,363,364]. Differently from other models of neurodegeneration, depletion of microbiota through either GF conditions or antibiotic use exacerbates the disease phenotype [362]. Consistent with these results, a large nested case-control study in the Swedish population revealed that the amount of antecedent antibiotic use may be positively associated with ALS risk [365]. 

Not surprisingly, GM changes result in major shifts in microbial components and metabolites produced by gut bacteria. A comprehensive study using a multimodal omics technology in animal models showed that manipulation of specific bacterial species may modulate outcomes in diseased mice, such that *Akkermansia muciniphila* ameliorates whereas *Ruminococcus torques* and *Parabacteroides distasonis* exacerbate ALS symptoms [362]. *Akkermansia muciniphila* improves survival by increasing levels of nicotinamide, involved in mitochondrial function and removal of toxic radicals in serum and cerebrospinal fluid of ALS mice [362]. The same study also revealed an aberrant nicotinamide metabolism in ALS patients, offering a potential translation of these findings to humans. The rise in harmful bacteria implicates the development of an inflammatory environment in gut, witnessed in both ALS patients and mice [364,366]. This leads to leakiness of the IEB and consequent higher exposure to endotoxins. Indeed, Zhang et al. showed that LPS is elevated in plasma of ALS patients, also overcoming levels observed in AD [367], which favors neuroinflammation through microglia activation. Another link between microbiota and ALS was reported by Burberry et al., who showed that a microbial environment enriched in pro-inflammatory bacteria elicits an exaggerated systemic and neural inflammation in *C9orf72* knockout mice, suggesting that *C9orf72* is implicated in the modulation of inflammatory responses elicited by altered GM both in- and outside the CNS [368]. 

A potential role for TDP-43 in the gut has not been investigated as for Aβ or α-syn, but clues may be caught between the lines. Rates of constipation in ALS may reach 30% of cases, which has been explained by the increased immobility occurring during disease progression [369]. Nonetheless, experimental studies on TDP-43 transgenic mice revealed a prominent, unexpected gut dysfunction characterized by intestinal dysmotility, loss of enteric neurons and epithelial alterations [370,371]. Such findings precede the development of motor symptoms, leading to death before the full motor phenotype becomes evident. Pathological studies on these animals described features of TDP-43 pathology in the mucosa and submucosa of colonic tissues, suggesting that enteric neurons may be susceptible to TDP-43 proteinopathy. 

Current evidence justifying the use of probiotics in ALS is scarce [359]. Nonetheless, promising results were published by Zhang et al., who showed that supplementation of butyrate restored gut integrity, microbial composition and prolonged survival in SOD1^G93A^ mice, suggesting that targeting SCFAs may be an additional strategy for therapeutic intervention [372].

## 5. Future Perspectives

Upon the new scenarios that await to be unraveled, open issues and methodological challenges need to be addressed. Analysis of the available evidence suggests that diet, microbiome and metabolic disorders affect the risk of neurological disorders upon a long-term exposure traced back at least to mid-life. The consequences of 10 to 20 years of bad dietary habits and dysbiosis may significantly hasten neuroinflammation and subsequent neuronal death. Only recently, experimental studies assessed the role of accumulated burden of environmental risk factors on the brain. Prolonged exposure to five peripherally induced risk factors (LPS, social stress, diabetes, high cholesterol diet, food contamination with heavy metals) led to memory impairment and cerebral amyloid angiopathy in aged mice, unraveling the contribution of these factors to AD-related pathology [373]. Among them, chronic endotoxemia may damage cerebral vascularization and prime microglia towards a neuroinflammatory phenotype [374]. In addition, gut-derived stimuli may also act as triggers of progressive neurodegeneration. PINK -/- mice show molecular signs of PD but fail to display a pathologic phenotype. Instead, when PINK -/- mice are exposed to intestinal infection by Gram-negative bacteria, progressive loss of dopaminergic neurons and PD-like motor abnormalities are observed [375], representing a striking example of how environmental risk factors and genetic susceptibility combine to set out the final outcome. Whether such complex modeling can be applied also to AD and ALS warrants further investigations. 

Dietary habits in humans are complex, displaying high variability in richness and composition. and the mutual interactions between different nutrients may result in null or potentiating effects, highlighting the need for higher discriminatory power to recognize independent and synergic effects. In such a scenario, the massive network that can be generated by the interactions of thousands of nutrients, metabolites, microbial products, and host-derived factors cannot be disentangled only by hypothesis-driven experimental studies and thus needs untargeted, non-biased approaches. Currently, machine learning and integrated multi-omics (genomics, transcriptomics, proteomics, lipidomics, epigenomics, metabolomics and so on) may be helpful to overcome such problems, and represent valid tools to identify patterns of harmful habits, to establish cause–effect relationships and to avoid confounder effects. In this regard, Samieri et al. recently reported that in France, food networks characterized by charcuterie, foods typical of French southwestern diet and snack foods, predispose to dementia [376]. Bi-directional interactions with microbiota and peripheral metabolism add another layer of complexity to the metabolomic status. For instance, polyphenols and ω-3 PUFAs may be beneficial to neurons through the stimulation of a healthy GM, which may further enhance their effect by generating nutrient-derived metabolites [377,378]. Meng et al. used systemic nutrigenomics to elucidate how ω-3 PUFAs counteract the harmful effects of fructose in brain rats [379], providing rational support for targeted dietary interventions in humans. Recent studies applying these approaches successfully identified key factors involved in PD pathogenesis [251]. This hold promises for future applications also in AD and ALS. 

Furthermore, the advantages offered by novel experimental models (co-cultures, 3D cultures, organoids, CRISPR/Cas9 gene editing) may aid in simulating these network systems for better analysis. For instance, organ-on-a-chip modeling was recently proposed as a candidate tool for future investigation of the gut–brain axis [380]. The system consists of chips serially connected through microfluidic devices. Each chip contains a mini organ, such as the microbiota, gut, blood barrier and the brain. In this way, detailed assessment of microbiota-associated changes can be investigated through the analysis of the secretome. 

Clinical interventions aimed at a healthy diet and microbiome have been carried out in recent years, still showing limited results. Significant protection may be obtained only if applied during long prodromal phase of neurodegenerative diseases, supporting its role for prevention rather than treatment. Indeed, clinical trials targeting multiple risk domains (FINGER, MAPT, preDIVA), including nutritional guidance, significantly slowed slopes of cognitive decline in the elderly population, especially on high-risk individuals [2]. At the same time, large observational studies are ongoing to comprehensively assess individuals predisposed to PD and AD and identify personalized paths for risk management [381,382]. Still, dietary interventions may be helpful for symptomatic relief and may assist main drug therapies by improving their pharmacokinetic and adverse effects’ profile [383]. Research on the GM–brain axis is essential to boost development of better neuroprotective probiotics. Commercially available products are mainly composed of *Lactobacillus*, *Bifidobacterium,* and related bacterial species, but it is reasonable to doubt whether these compositions work well for all human diseases. As a warning, recent studies highlighted that improper administration of probiotics in healthy individuals may elicit cognitive dysfunction as a side effect [384,385]. Finally, FMT may be an alternative strategy to tackle dysbiosis in neurodegenerative diseases. Anecdotal reports of FMT use in PD have been reported, which prompted the design of randomized clinical trials [386]. In addition, a multicenter trial evaluating the efficacy of FMT in ALS (FETR-ALS) is ongoing, which will assess possible changes in gut microbiota and effects on clinical outcomes [387]. 

## 6. Conclusions

The present review highlighted the most relevant advances in the relationship between neurodegenerative diseases, nutrition, and microbiota. In particular, recent findings suggest that AD, PD, and ALS share common features of gut alterations, characterized by dysbiosis, increased barrier permeability and inflammation, leading to a chronic low-grade endotoxemia and inflammation. Such a pathological state, detected in the pre-symptomatic or early phase of the disease, participates in progressive neurodegeneration by damaging neural vasculature, exacerbating aggregation of prion-like proteins, and promoting aberrant neuroinflammation. Nonetheless, the relative contribution of these mechanisms may vary, as it seems relevant for PD while its role in ALS still need to be defined.

Besides common factors, disease-specific relationships exist, as features of metabolic syndrome may predispose to AD and PD but appear protective against ALS. Midlife is a critical time period in which dietary habits and metabolic disorders significantly increase the risk of neurodegeneration in the following 10-20 years, exhibiting potential for targeted prevention strategies in the general population. Notwithstanding this, therapeutic options applied in the management of metabolic disorders failed to show clear neuroprotective effects.

To date, interventions focused on nutritional habits and the microbiome showed promising results, although robust evidence is still lacking. Application of novel technologies and integrated, multidisciplinary studies combined to the innovative experimental models may uncover the world beneath to foster novel therapeutic strategies and support evidence-based public health recommendations against neurodegenerative diseases.

## Figures and Tables

**Table 1:** Effects of metabolic disorders, diet, and gut microbiota on Alzheimer’s disease (AD).

	Harmful	Protective
Metabolism		
Obesity	Human: risk in midlife. Mice: memory loss, Aβ deposition and gliosis.	
Diabetes	Human and mice: defects in insulin/IGF-1 pathway in brains.	
Dyslipidaemia	Human: risk in midlife. Mice: correlates with burden of Aβ pathology.	
MAFLD	Human: impaired cognition. Mice: AD-like pathology.	
**Diet**		
Fat intake	Human: saturated fat. Mice: high-fat diet worsens severity in AD-mice.	Human and mice: ω-3 PUFAs protect neurons, reduce inflammation and vascular damage.
Antioxidants		Human and mice: counteract ROS production, lipid peroxidation, DNA damage.
Dietary patterns		Human: MeD. Human and mice: caloric restriction.
**Microbiota**		
Composition	Opportunistic gram-negative bacteria	SCFA-producing bacteria.
Mechanisms	Endotoxin exposure, IEB permeability, gut inflammation.	SCFAs supply to gut and brain
Gut-brain axis	Aβ deposition in the gut of animal models. Bacterial amyloids may aid in cross-seeding. Gut-to-brain transport after prolonged time.	
Gut microbiota-based Therapy		Improve metabolic markers. Efficacy on cognition is limited.

**Table 2:** Effects of metabolic disorders, diet and gut microbiota on Parkinson’s disease (PD).

	Harmful	Protective
**Metabolism**		
Obesity	No relationship found	
Diabetes	Increase risk and clinical severity Mitochondrial dysfunction as a common mechanism	
Dyslipidaemia	Exacerbation of motor deficits in animal models.	Decrease risk and progression in humans
MAFLD	Unclear association DJ-1 dysfunction may be a potential common mechanism	
**Diet**		
Fat intake	Uncertain association with saturated fats High-fat diet worsen motor symptoms and neuronal loss in mice.	Ω-3 PUFAs
Antioxidants		Uric acid decrease risk and progression
Food	Milk (unknown mechanism).	Coffee (A2a receptor antagonist).
Dietary patterns		MeD Caloric restriction in humans and animals.
**Microbiota**		
Composition	Opportunistic gram-negative bacteria Lactobacillus, Bifidobacterium, Akkermansia	SCFA-producing bacteria.
Mechanisms	Endotoxemia, IEB permeability, gut inflammation. Sulfite production	SCFAs supply.
Gut-brain axis	α-syn deposition in the gut (humans and animals). Bacterial amyloids enhance α-syn aggregation. Gut-to-brain transport demonstrated.	
Gut microbiota-based Therapy		Probiotics improve constipation and metabolic markers. Potential to treat other non-motor symptoms (e.g., depression).

**Table 3:** Effects of metabolic disorders, diet and gut microbiota on amyotrophic lateral sclerosis (ALS).

	Harmful	Protective
**Metabolism**		
Obesity		Decrease risk and progression Counteracts ALS hypermetabolism
Diabetes	Mixed effects (depends on ethnicity) IGT observed in patients Different mechanisms form classic diabetes	
Dyslipidaemia	Increased risk of disease	Decreased mortality Early switch to lipid metabolism in motor neurons and muscles may be an early compensatory mechanism
MAFLD	Hepatic steatosis frequent finding Unknown significance	
**Diet**		
Fat intake	ω-3 PUFAs may exert a double-edge role	Associated with longer survival
Antioxidants		Lowers disease risk Intake correlates with higher functional scores Co-supply with ω-3 PUFAs may show synergic effects
Dietary patterns		Human: MeD Mice: caloric restriction worsens disease severity
**Microbiota**		
Composition	Opportunistic gram-negative bacteria	SCFA-producing bacteria
Mechanisms	Endotoxemia, IEB permeability, gut inflammation Deletion of C9orf72 exaggerates systemic immune response	Nicotinamide and SCFAs levels
Gut-brain axis	TDP-43 deposition in the gut of animal models Gut-to-brain transport not assessed	
Gut microbiota-based Therapy		Mice: SCFAs alleviate motor symptoms

## Abbreviations

AD	Alzheimer’s disease
AHR	Aryl hydrocarbon receptor
ALS	Amyotrophic lateral sclerosis
APOB	Apolipoprotein B
APOE	Apolipoprotein E
APP	Amyloid precursor protein
Aβ	Amyloid-β
BBB	Blood–brain barrier
BDNF	Brain-derived neurotrophic factor
BMI	Body Mass Index
C9orf72	Chromosome 9 open reading frame 72
CIDP	Chronic inflammatory demyelinating polyneuropathy
CNS	Central nervous system
DHA	Docosahexaenoic acid
EAE	Experimental autoimmune encephalomyelitis
ENS	Enteric nervous system
EPA	Eicosapentaenoic acid
FA	Fatty acids
FGF21	fibroblast growth factor 21
FMT	Fecal microbial transplantation
FUS	Fused in sarcoma
GCS	Glucosylceramide synthase
GF	Germ-free
GLP-1	Glucagon-like peptide-1
GM	Gut Microbiota
HCAR2	Hydroxycarboxylic acid receptor 2
IEB	Intestinal epithelial barrier
IGF-1	Insulin-like growth factor-1
IL-1β	Interleukin-1β
IL-6	Interleukin-6
LDL-C	Low density lipoprotein-cholesterol
LPS	Lipopolysaccharide
LRRK2	Leucine-rich repeat kinase 2
MAFLD	Metabolic dysfunction-associated fatty liver disease
MeD	Mediterranean Diet
MS	Multiple sclerosis
MTHFD1	Methylenetetrahydrofolate dehydrogenase-1
NF-kB	Nuclear factor kappa-light-chain-enhancer of activated B cells
NLRP3	Nucleotide-binding oligomerization domain, leucine rich repeat and pyrin domain containing 3
PARK7	Parkinson disease protein 7
PD	Parkinson’s disease
PGC1α	Peroxisome proliferator activated receptor gamma coactivator 1-α
RBD	Rapid eye movement sleep behaviour disorder
ROS	Reactive oxygen species
SCFA	Short-chain fatty acids
SLC2A9	Solute carrier family 2 member 9
SNCA	α-synuclein (gene)
SOD1	Superoxide dismutase 1
T2D	Type 2 diabetes
TARDBP	TAR DNA binding protein-43
TDP-43	Transactive response DNA-binding protein 43
T_h_	T helper cells
TLR	Toll-like receptor
TNF-α	Tumor necrosis factor-α
T_reg_	T regulatory cells
WT	Wild type
α-syn	α-synuclein (protein)
ω-3 PUFA	Omega-3 polyunsaturated fatty acids
